# Biomechanics of the JCT and SC Inner Wall Endothelial Cells with Their Basement Membrane Using 3D Serial Block-Face Scanning Electron Microscopy

**DOI:** 10.3390/bioengineering10091038

**Published:** 2023-09-04

**Authors:** Alireza Karimi, Reza Razaghi, Mary J. Kelley, Ted S. Acott, Haiyan Gong

**Affiliations:** 1 Department of Ophthalmology, Casey Eye Institute, Oregon Health & Science University, Portland, OR 97208, USA; razaghireza@outlook.com (R.R.); kelleyma@ohsu.edu (M.J.K.); acott@ohsu.edu (T.S.A.); 2 Department of Biomedical Engineering, Oregon Health & Science University, Portland, OR 97208, USA; 3 Department Integrative Biosciences, School of Dentistry, Oregon Health & Science University, Portland, OR 97208, USA; 4 Department Chemical Physiology & Biochemistry, School of Medicine, Oregon Health & Science University, Portland, OR 97208, USA; 5 Department of Ophthalmology, Boston University School of Medicine, Boston, MA 02118, USA; hgong@bu.edu; 6 Department of Anatomy and Neurobiology, Boston University School of Medicine, Boston, MA 02118, USA

**Keywords:** trabecular meshwork, juxtacanalicular tissue, Schlemm’s canal, outflow resistance, fluid–structure interaction

## Abstract

Background: More than ~70% of the aqueous humor exits the eye through the conventional aqueous outflow pathway that is comprised of the trabecular meshwork (TM), juxtacanalicular tissue (JCT), the inner wall endothelium of Schlemm’s canal (SC). The flow resistance in the JCT and SC inner wall basement membrane is thought to play an important role in the regulation of the intraocular pressure (IOP) in the eye, but current imaging techniques do not provide enough information about the mechanics of these tissues or the aqueous humor in this area. Methods: A normal human eye was perfusion-fixed and a radial wedge of the TM tissue from a high-flow region was dissected. The tissues were then sliced and imaged using serial block-face scanning electron microscopy. Slices from these images were selected and segmented to create a 3D finite element model of the JCT and SC cells with an inner wall basement membrane. The aqueous humor was used to replace the intertrabecular spaces, pores, and giant vacuoles, and fluid–structure interaction was employed to couple the motion of the tissues with the aqueous humor. Results: Higher tensile stresses (0.8-kPa) and strains (25%) were observed in the basement membrane beneath giant vacuoles with open pores. The volumetric average wall shear stress was higher in SC than in JCT/SC. As the aqueous humor approached the inner wall basement membrane of SC, the velocity of the flow decreased, resulting in the formation of small eddies immediately after the flow left the inner wall. Conclusions: Improved modeling of SC and JCT can enhance our understanding of outflow resistance and funneling. Serial block-face scanning electron microscopy with fluid–structure interaction can achieve this, and the observed micro-segmental flow patterns in ex vivo perfused human eyes suggest a hypothetical mechanism.

## 1. Introduction

**Figure 1:**
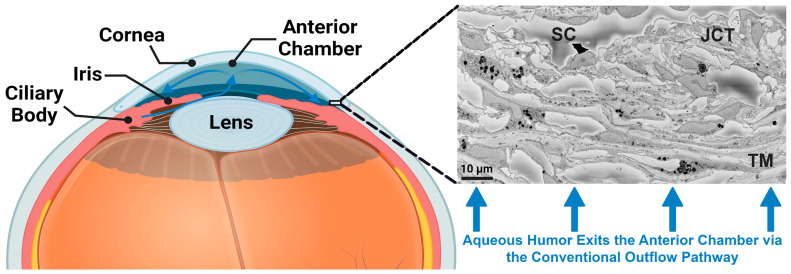
Anterior segment of the human eye. The aqueous humor is secreted from the ciliary body at a rate of 2–3 µL/min and flows into the anterior chamber. More than ~70% of the aqueous humor then drains via the conventional outflow pathway that is located in the corner of the cornea and iris. The conventional aqueous outflow pathway is comprised of the TM, JCT, SC inner wall endothelial cells, SC, collector channels, and episcleral venous drainage system.

The aqueous humor is secreted by the ciliary body at the rate of 2–3 µL/min and flows into the anterior chamber as shown in Figure 1. The majority of the aqueous humor then exits the eye through a network of structures, namely the conventional aqueous outflow pathway, that is comprised of the trabecular meshwork (TM), juxtacanalicular tissue (JCT), inner wall endothelium of Schlemm’s canal (SC) and exits through collector channels and the venous drainage system (Figure 1) [1,2,3,4,5,6]. In the normal eye, the JCT and basement membrane of the SC inner wall are the major sites of the resistance to aqueous humor outflow in this pathway [7,8,9]. The biomechanical properties of the outflow tissues also play a key role in intraocular pressure (IOP) regulation [9,10,11,12,13] through a dynamic, two-way, fluid–structure interaction (FSI) coupling between the outflow tissues and the aqueous humor [14,15,16,17,18,19,20,21].

The main areas of the TM where aqueous humor outflow resistance occurs are believed to be the extracellular matrix (ECM) of the JCT and basement membrane of the inner wall endothelial cells of SC, and modified by the pores of the inner wall endothelial cells of SC, which was previously thought to be responsible for around 10% of the resistance [22]. However, this study did not consider the funneling effect. Mark Johnson showed that the interaction between the pores of the inner wall of SC and the underlying JCT can lead to a significant increase in outflow resistance through a funneling effect. This effect confines the aqueous flow to the JCT areas located near the pores of the inner wall, creating a funneling pattern of outflow. Therefore, the pores may play a role in regulating the resistance in this region [23]. An abnormal ECM generation in the JCT or basement membrane of SC may cause a disruption in the funneling through the inner wall pores, leading to an increase in the outflow resistance [24]. Recent studies have shown that excessive ECM formation in the JCT and trabecular beam thickening of primary open-angle glaucoma (POAG) eyes compared to normal human eyes may be responsible for abnormally high outflow resistance in this region [25], as has been observed in POAG eyes [26]. The porous structure of the JCT, as well as the number and size of pores in the inner wall of SC, are significant factors that contribute to the overall outflow resistance, as they facilitate the flow from the homogenously porous JCT structure to the low porosity inner wall endothelium and its greater basement membrane, namely funneling [17,23]. This is because the basement membrane is discontinuous in normal eyes.

Mark Jonson’s calculations showed that the effective JCT flow resistance is correlated to the SC inner wall pore density, which has important implications for the pathogenesis of POAG [23]. Allingham also showed a significant decrease in the number of pores in the SC inner wall of POAG eyes [27]; thus, the funneling in POAG eyes may look quite different compared to normal eyes. In order to maintain the funneling effect, it is crucial to maintain the connection between the inner wall and JCT. If this connection is lost, the flow through the JCT to the inner wall may be disrupted, causing the flow through the JCT to become uniform [17]. Our group used dynamic FSI and computational fluid dynamics to model the funneling through the geometrically simplified pores of the inner wall endothelium of SC [13]. Our findings revealed that eyes with more compliant outflow tissues exhibit consistent funneling of fluid through the pores of the inner wall of SC. In scenarios where the outflow tissues undergo stiffening, either due to aging or the presence of glaucoma, the process of funneling can become disrupted. This alteration may lead to the formation of small eddies within the JCT area, while the flow is still laminar (Reynolds number ~70) [13]. This could be a reason for an abnormally high outflow resistance in the glaucoma eyes. In our previous models, we used a simplified representation of the pores in the inner wall of SC, assuming that they were uniformly distributed with a diameter and thickness of 1.3 µm and 835 pores/mm^2^, respectively [16,28]. However, it has been demonstrated that the actual geometry of the pores in the inner wall of SC is more intricate [29,30]. To address this limitation, we utilized serial block-face scanning electron microscopy (SBF-SEM) images [29,30] to create a precise and more realistic 3D finite element (FE) model of the JCT and inner wall endothelial cells with their basement membrane, which incorporates the complex features of the intertrabecular spaces, pores, and giant vacuoles. This is the most accurate 3D structure of the JCT and inner wall endothelial cells of SC with their basement membrane to date developed by Haiyan Gong’s team. By developing this detailed model, we can better investigate the fluid dynamics of the aqueous humor within this region while considering the active biomechanical interactions between the outflow tissues and the aqueous humor.

Haiyan Gong’s research team created a technique using a two-color fluorescent tracer to examine the fluid dynamics of the aqueous humor in bovine eyes. The technique involved using fluorescent microbeads with a diameter and density of 0.5 µm and 0.002% *v*/*v*, respectively. In a 500 mL solution, 0.002% *v*/*v* would mean that there are 0.02 mL of the solute and 499.98 mL of the solvent. They measured the outflow facility at two different baseline IOPs of 30 mmHg that were then lowered to 7 mmHg [31]. Although this method provided valuable insights into changes in the aqueous humor outflow pattern with changes in IOP, there is still a gap in our understanding of the local biomechanical behavior of the JCT, and inner wall endothelial cells of SC with their basement membrane. Regions with varying aqueous humor velocity in the outflow pathway may represent low- and high-flow regions that are correlated with the biomechanical properties of the outflow tissues [9,32]. Therefore, a more comprehensive experimental–computational workflow that provides a wider range of information on the regional biomechanics and hydrodynamics of the aqueous humor could significantly enhance our understanding of aqueous humor outflow resistance and IOP regulation in both healthy and glaucomatous human eyes.

This study utilized an eye from a healthy donor with high-flow regions labeled with fluorescent microspheres and perfusion fixation at a baseline IOP of 7 mmHg. Tissue wedges of the TM, including SC, were imaged using SBF-SEM. The resulting images were segmented to generate a 3D FE model of the JCT and inner wall endothelial cells with their basement membrane, with the intertrabecular spaces, pores, and giant vacuoles replaced by the aqueous humor [29,30]. The FSI with multi-material arbitrary Lagrangian–Eulerian (MM-ALE) as a sub-formulation was used to model the biomechanical motion of the outflow tissues (Lagrangian element) due to the aqueous humor outflow (Eulerian element).

The primary objective of this study was to establish a robust and effective experimental–computational workflow for investigating the biomechanics of the inner wall endothelial cells of SC with their basement membrane and JCT in the conventional aqueous outflow pathway. This area of research suffers from a lack of sufficient information due to limitations in current imaging techniques. In this study, we have utilized the serial block-face scanning electron microscope images from a single human donor eye since the primary goal was to develop an experimental–computational workflow to study the biomechanics of the JCT and inner wall endothelial cells with their basement membrane, but we aim to extend our modeling to a larger and more diverse cohort of both healthy and glaucoma-affected human eyes. Herein, we specifically chose slices where basement membrane discontinuities, the apparent pathway of aqueous humor through the ECM outflow resistance, were absent. This provided the model with an intact outflow resistance and no easy path of egress. This should produce stresses as would be found due to IOP increasing. The basement membrane is a specialized ECM that underlies the endothelial cells of SC. It consists of various proteins, glycoproteins, and proteoglycans that form a complex network. The basement membrane contributes to outflow resistance primarily through its mechanical properties and composition. It affects the porosity and permeability of the inner wall of SC, thereby influencing the ease with which aqueous humor can flow through the conventional outflow pathway. In conditions like glaucoma, changes to the basement membrane, such as thickening or stiffening, can further increase outflow resistance. The basement membrane’s interaction with other components of the outflow pathway, such as the endothelial cells of SC and the extracellular matrix of the JCT, also plays a role in regulating outflow resistance. This workflow may significantly enhance our understanding of the mechanisms modulating outflow resistance in healthy human eyes and the hydrodynamics of funneling in the JCT and inner wall endothelial cells with their basement membrane eventually leading to a better understanding of the effects of different IOP-lowering drugs in altering the rheology and biomechanics of the outflow pathway in the future. However, the mechanisms underlying their effects on the mechanical properties of the TM and the dynamics of aqueous humor flow through the outflow pathway remain unclear. Understanding these mechanisms is crucial for developing more effective drugs for glaucoma treatment.

## 2. Materials and Methods

### 2.1. Human Donor Eyes, Ocular Perfusion and Fixation, Global Imaging, and Serial Block-Face Scanning Electron Microscopy

A human donor eye of a 32-year-old (female, Caucasian descent) without any known history of ocular diseases was obtained within 24 h postmortem from the Miracles in Sight Eye Bank (Winston-Salem, NC, USA). The eye was confirmed to be grossly normal under a dissecting microscope and was used in accordance with the guidelines regarding use of human subjects and tissues as outlined in the Declaration of Helsinki.

The whole globe was perfused using pressure-controlled hydraulic pumps with Dulbecco’s phosphate-buffered saline (pH 7.3; Invitrogen, Grand Island, NY, USA) with 5.5 mM D-glucose (collectively referred to as GPBS) for 30 min to establish a stable baseline facility at constant pressure of 7 mmHg [30]. Fluid perfusion was then changed and the eye was perfused with a fixed volume (200 µL; 1:1000 dilution in GPBS) of red fluorescent tracers (size: 200 nm; Catalog number: F8810; Thermo Fisher Scientific, Waltham, MA, USA) to label the high and low flow regions.

Following this, the eye was perfusion-fixed with modified Karnovsky’s fixative (consisting of 2.5% glutaraldehyde and 2% paraformaldehyde in 0.1-M sodium phosphate buffer with a pH of 7.3) for 30 min. Afterward, a small cut of approximately 5 mm was gently made along the equator of the eye, and it was then immersed in the same fixative overnight.

To visualize the outflow pattern of the entire limbus, the eye was hemisected into anterior and posterior segments [33]. The iris, ciliary body, cornea (10 mm trephine), vitreous, and excess conjunctiva were removed. The anterior segments were imaged en Face on both the TM and episcleral surfaces using a 300 mm lens on a 4000 MP VersaDock imaging system with fixed exposure time. Tissue wedges of the TM, including SC and small part of sclera, were then dissected from flow-type areas (high-, low-, and non-flow regions) guided by the global images. The size of the tissue wedges was 2 mm × 2 mm × 2 mm in width, length, and thickness, respectively [30]. In this study, tissue wedges from the high-flow region of the human donor eye were selected for analysis.

**Figure 2:**
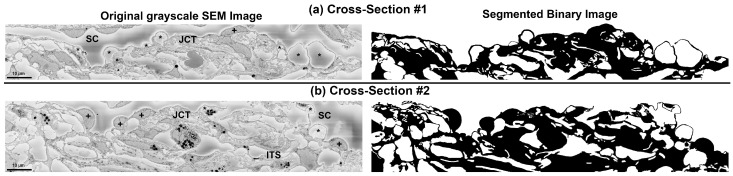
Original grayscale serial block-face scanning electron micrograph (SEM) images for the cross-sections (**a**) #1 and (**b**) #2. Schlemm’s canal (SC) with inner wall cell nuclei (+) and the giant vacuoles (*). ITS = intertrabecular space; JCT = juxtacanalicular connective tissue. The boundary of the tissues in the original grayscale SEM images was segmented to generate binary images for volume mesh reconstruction.

The tissue wedge was sent to Cleveland Clinic (Cleveland, OH, USA) for processing [34]. Tissue was post-fixed with OsO4/K ferrocyanide and thiocarbohydrazide and en-bloc stained with uranyl acetate and lead aspartate. Tissue was dehydrated with a series of ethanol dilutions and embedded in Epon resin. The regions of interest (including the SC and TM) in each block were imaged using a Zeiss Sigma VP SBF-SEM equipped with a Gatan 3View (Gatan, Inc., Pleasanton, CA, USA) in-chamber ultramicrotome stage and low-kV backscattered electron detectors that were optimized for 3View systems. After each image was taken of the block face, the automated ultramicrotome cut a section, and then another image was taken of the block face until the entire block was cut through. Pixel size was 0.0101-μm × 0.0101-μm, and section thickness was 0.13-μm. The original grayscale SBF-SEM images from two different cross-sections are shown in Figure 2 (left panel). It is worth mentioning that it has been reported that there is no significant correlation between I-pore or B-pore density and post mortem time [30].

### 2.2. Segmentation, 3D Finite Element Model, Material Model, and Fluid–Structure Interaction

The segmentation and volume meshing algorithms are fully explained in our prior publications [12,13,25,35,36,37]. In the chosen SBF-SEM images [30], the JCT, SC inner wall endothelial cells with their basement membrane, pores, and giant vacuoles were identified and isolated as binary or black and white images, as depicted in Figure 2 (right panel). Firstly, the grayscale images were converted to binary images, after which a semi-automatic custom MATLAB algorithm (R2022b, Mathworks, Natick, MA, USA, licensed to OHSU academic user) was employed to identify the tissue boundaries and eliminate the grayscale-to-binary artifacts [38]. In the context of this study, the black dots observed near the tissue boundaries in the JCT or SC inner wall are referred to as artifacts, caused by shading in the grayscale SBF-SEM images.

**Figure 3:**
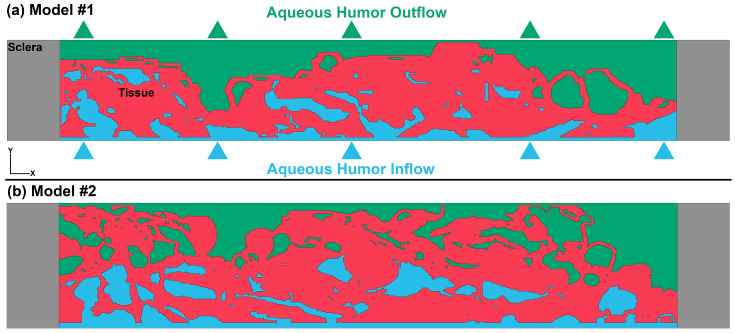
Finite element volume mesh of the SBF-SEM image with the thickness of 0.26 µm for the image set of (**a**) #1 and (**b**) #2. Finite element model of the JCT and SC inner wall endothelial cells and their basement membrane (red). The flow (Eulerian part) was separated into two different parts, including the region with a pressure of 7 mmHg (blue) and 0 mmHg (green). The flow was coupled with the JCT and SC inner wall endothelial cells with their basement membrane (Lagrangian part) using multi-material ALE formulation. The lateral sides of the model were constrained with the sclera with a length of 12 µm. The JCT, SC inner wall endothelial cells with their basement membrane and sclera were modeled as linear elastic with constant stress solid element formulation. The aqueous humor was modeled as an incompressible viscous fluid with one-point Eulerian formulation.

To create a three-dimensional (3D) mesh representation of the binary SBF-SEM images that were segmented (as shown in Figure 2, right panel), we utilized an image-to-mesh algorithm that we recently developed [37,39]. The resulting volume mesh encompassed the JCT and SC inner wall endothelial cells with their basement membrane, including the intertrabecular spaces that were filled with aqueous humor, as depicted in Figure 3. To create a microstructural FE mesh of the JCT/SC complex, a larger parent volume mesh with 8-noded hexahedral elements was first reconstructed with an edge length of approximately 0.25 µm. This mesh was then aligned with the microstructural FE mesh of the JCT/SC complex using the same coordinate system. However, it should be noted that the element edge length of the reconstructed FE mesh was larger than the voxel resolution (0.0101 µm) of the SBF-SEM images. In order to ensure that the resulting strains and hydrodynamics in the outflow tissues were independent of the number of elements, mesh density analyses were conducted [25,36,37,40]. This was conducted to verify that the mesh density was appropriate and that the results were not affected by the mesh size. By conducting these analyses, it was possible to ensure that the resulting FE model accurately represented the microstructure of the JCT/SC complex and that the simulated mechanical behavior was reliable. The meshing algorithm was then used to separate the parent volume mesh to the JCT/SC complex with interspersed aqueous humor [39,41]. To ensure the accuracy of the FE models, a comparison was made between the 3D reconstructed microstructure of the JCT/SC complex and the SBF-SEM images at several cross-sections (approximately 5) using boundary overlay [36,39]. To provide a “physiological” boundary condition to tether the model and control the axial and transversal movements of the JCT/SC complex, a sclera with a thickness and length of 0.26 µm and 12 µm, respectively, in the X direction was included as a boundary tissue in both models #1 and #2 as shown in grey in Figure 3.

The JCT and SC inner wall endothelial cells with their basement membrane were modeled as nearly incompressible elastic material with the elastic modulus, Poisson’s ratio, and density of 3.05 kPa (high-flow region after 24 h perfusion), 0.495, 700 kg/m^3^, respectively [32]. The sclera also was modeled as nearly incompressible elastic material with the elastic modulus, Poisson’s ratio, and density of 2.54 MPa, 0.495, and 1000 kg/m^3^, respectively [42].

The FSI formulations are fully explained in our prior publications [13,25,36,37]. The MM-ALE (mixed mesh arbitrary Lagrangian–Eulerian) method was used to define the solid and fluid domains in the JCT/SC complex (Lagrangian) and aqueous humor (Eulerian), respectively [43,44]. MM-ALE implemented through the Ansys/LS-DYNA software, employs automatic mesh refinement and allows for Lagrangian and Eulerian mesh motions to be incorporated into a single framework [45,46]. The MM-ALE defines the appropriate ALE material groupings for interface reconstruction. The flow during ocular perfusion has similar properties to that of the aqueous humor and was simulated as homogeneous, Newtonian, and viscous [47], with the density and dynamic viscosity of 1000 kg/m^3^ and 0.7185 × 10^−3^ Pa·s [48], respectively. The inflow and outflow regions for the aqueous humor are illustrated in Figure 3. The flow also had the Gruneisen equation of state. The Gruneisen equation of state with cubic shock velocity–particle velocity defines pressure for compressed material as [49,50]:

(1) $$p=\frac{\rho_{0}C^{2}\mu [1+\left(1-\frac{\gamma_{0}}{2}\right)\mu -\frac{a}{2}\mu^{2}]}{\left[1-\left(S_{1}-1\right)\mu -S_{2}\frac{\mu^{2}}{\mu +1}-S_{3}\frac{\mu^{3}}{{\left(\mu +1\right)}^{2}}\right]}+(\gamma_{0}+\alpha \mu )E$$

where E is the internal energy per initial volume, C is the intercept of the $u_{s}-u_{p}$ curve, $S_{1}$, $S_{2}$, and $S_{3}$ are the coefficients of the slope of the $u_{s}-u_{p}$ curve, $\gamma_{0}$ is the Gruneisen gamma, and a is the first-order volume correction to $\gamma_{0}$. Constants, C, $S_{1}$, $S_{2}$, $S_{3}$, $\gamma_{0}$, and α, are user-defined input parameters for the aqueous humor. Herein, the constants were defined as C=1480 m/s, $S_{1}=2.56$, $S_{2}=-1.98$, $S_{3}=0.226$, $\gamma_{0}=0.5$, and α=0 [13,36,37]. The compression is defined in terms of the relative volume, V, as:

(2) $$\mu =\frac{1}{V}-1$$

where the initial relative volume of the flow in our simulations was defined as one (1), which means the flow was initially incompressible. A constrained Lagrange in solid coupling algorithm was used to model the FSI structure. The coupling sub-algorithm was penalty coupling (without erosion) with tension and compression formulations. The penalty factor, which is a scale factor for scaling the estimated stiffness of the interacting (coupling) system is used to compute the coupling forces to be distributed on the solid and fluid parts. The penalty factor here is set to 0.1, which provides a strong coupling between the flow and solid with no leakage factor.

Due to computational limitations, we were unable to interconnect the pores in 3D in the SBF-SEM images, resulting in artificial discontinuity in the flow within the JCT/SC complex. To address this issue, we reconstructed another component that encompasses the entire tissue FE mesh with MM-ALE method. In other words, the tissue was considered to be sunk in the aqueous humor, which flowed into the model from the anterior chamber. This helped us to interconnect the fluid across the JCT/SC complex and apply the same pressure (7 mmHg, the component with blue color) in the desired regions where the aqueous humor actively interacts with the solid JCT (including the JCT matrix and cells) and SC inner wall endothelial cells with their basement membrane. This approach also provides a physiologically relevant model despite being unable to include the entire set of SBF-SEM images. In model #2, we included a number of spaces that were on the anterior chamber side of the SC basement membrane at a pressure of 0, i.e., green. This was completed to look at the impact of outflow resistance further away from the SC cells and their basement membrane. We want to emphasize that the flow regions with the pressures of 7 mmHg (blue) and 0 mmHg (green) are just an initial condition of the model. Obviously, when the simulations begin, the inflow starts to elevate the pressure from 0 to 7 mmHg; thus, the pressure rises in all the regions including both the green and blue regions to reach 7 mmHg which is defined in our load boundary. Therefore, the blue and green regions should not be interpreted as static pressure distributions throughout the entire simulation, but rather as an initial condition that is modified by the flow and the resulting pressure distribution that develops during the simulation. This is important to keep in mind when interpreting the results of our model and understanding the dynamic nature of the pressure distribution within the system. The reason for including the 0 mmHg in a flow segment was for simplification and other values will be investigated in future studies. The resistance of the SC endothelial cells with their basement membrane cannot be fully modeled using FSI in this situation, thus, we have defined regions with 0 mmHg pressures to manipulate the regionalized resistance.

Two free surfaces of the model were subject to a symmetrical boundary condition to mimic the continuity of the network structure and provide a physiologically plausible model. Specifically, the translational degree of freedom in the Z direction and the rotational degree of freedom in the X and Y directions were constrained (Figure 3). The non-reflective boundary condition was used at the outlet boundary with a free boundary condition. This type of boundary condition is typically used on the outer boundaries of an analysis model with an infinite domain, such as a half-space, to prevent artificial stress wave reflections generated at the boundaries from re-entering the model and affecting the results. LS-DYNA calculates an impedance-matching function for all non-reflecting boundary segments based on the assumption of linear material behavior [45,46]. Without a non-reflective boundary condition in the outlet of the model, the aqueous humor would flow back and generate a pressure wave that pushes against the SC inner wall in the opposite direction (minus Y direction), which is probably not physiologically accurate. Therefore, it was important to define the non-reflective boundary condition to prevent this from happening. Additionally, a self-contact was defined between tissue elements to ensure that tissue deformation resulting from aqueous humor inflow or outflow would consider the contact between tissues.

A computer equipped with a 112-core Intel^®^ Xeon^®^ Gold 6258R CPU @ 2.70 GHz and 1 TB RAM was utilized to conduct the FSI simulation using dynamic explicit LS-DYNA. The loading was gradually applied over a period of 0.5 s with a time step of 0.001, resulting in 500 time steps. The FSI simulation required an average of approximately 205 h to complete on our workstation.

## 3. Results

**Figure 4:**
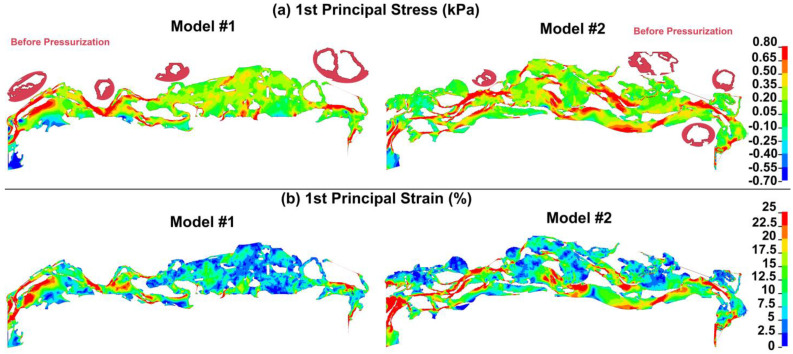
First principal or tensile (**a**) stress and (**b**) strain in models #1 and #2 at the aqueous humor pressure of 7 mmHg. The geometry of the model in different regions at the aqueous humor pressure of 0 mmHg is also shown in the inset next to each model. The model was based on an elastic material model and did not take into account the tissue’s tensile strength. Consequently, the model could not accurately simulate the failure or fracture of the tissue within the giant vacuoles and pores. The elements within these regions were subjected to a significant amount of tension, which could potentially lead to tissue failure. Similarly, the elements next to the sclera experienced significant tension due to their contact with a much stiffer tissue. Strain intensity scales are on the right side of the figures.

The first principal (tensile) stresses and strains in the JCT/SC complex are shown in Figure 4. The present study employs an elastic material model to analyze the mechanical behavior of tissues, which does not account for the tensile strength of the material. Consequently, under high tensile loads, the tissue elements may experience extensive stretching without undergoing failure. In particular, failure is a complex phenomenon that is not well captured by elastic material models. Tissues can fail in many ways, including fracture, tearing, and delamination, and the precise mode of failure can depend on the tissue structure, loading conditions, and other factors. Moreover, tissues often exhibit complex strain localization and damage accumulation, which can be difficult to model using simple elastic material models. To better understand tissue failure, more advanced models are needed that can capture the nonlinear and viscoelastic behavior of tissues, as well as their failure modes and damage accumulation. These models may include features such as strain softening, fracture mechanics, and damage evolution, and may require sophisticated computational techniques to simulate. However, it is reasonable to hypothesize that the regions where the elements undergo significant stretching may actually be susceptible to failure, particularly in the case of giant vacuoles, as illustrated in the insets of Figure 4. Larger stresses (~0.8 kPa) and strains (25%) occur in the SC endothelial cells with their basement membrane, particularly in the presence of open pores (Model#1, Figure 4). However, it should be noted that the failure might be very much geometry-dependent as some of Gong’s group data showed that giant vacuoles even at a higher IOP of 30 mmHg did not fail.

**Figure 5:**
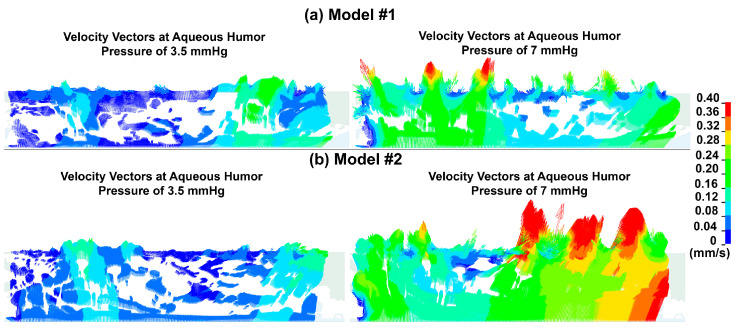
Velocity vectors at aqueous humor pressure of 3.5 and 7 mmHg in models (**a**) #1 and (**b**) #2. The direction of velocity vectors for aqueous humor across the JCT/SC complex changes during pressure elevation due to an active biomechanical interaction between elastic tissues and the incompressible aqueous humor. During certain stages of pressure elevation, small eddies are formed near the inner wall and SC. To illustrate the changing direction of velocity vectors as aqueous humor pressure increases, Supplementary Videos have been provided for both models #1 and #2.

The velocity vectors at aqueous humor pressures of 3.5 and 7 mmHg for models #1 and #2 are demonstrated in Figure 5. The elastic JCT/SC complex is actively involved in biomechanical interactions with the incompressible aqueous humor through FSI. The alteration of velocity vectors as a function of aqueous humor pressure elevation is observed, where the regions located immediately after the inner wall in SC exhibit smaller velocities. To accurately represent the changes in velocity vectors, only the regions far from the scleral boundary condition were considered, as the sclera could influence the aqueous humor hydrodynamics, leading to inaccurate results. We observed the occurrence of funneling in the close proximity of the inner wall of SC when the flow from the capacious intertrabecular spaces approached the basement membrane of the inner wall endothelial cells of SC. Supplementary Materials comprised of two videos (Supplementary Material Model #1 and #2) showing the velocity vectors across the aqueous humor in the JCT/SC complex are provided to enhance the representation of the velocity vector alterations in response to aqueous humor pressure elevation.

**Figure 6:**
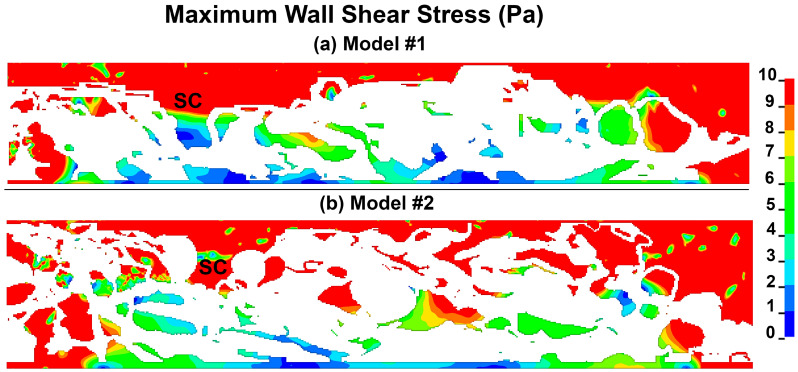
Maximum aqueous humor wall shear stress/cell surface shear stress at the pressure of 7 mmHg in models (**a**) #1 and (**b**) #2. The shear stress herein is only plotted on the surface of the model that is in interaction with the aqueous humor (flow).

The maximum aqueous humor wall shear stress or the shear stress in the SC endothelial cells across the JCT/SC complex at the aqueous humor pressure of 7 mmHg in models #1 and #2 are shown in Figure 6. Wall shear stress in a fluid refers to the frictional force that acts at the interface between a fluid and a solid surface (such as a wall), causing the fluid to move or flow. It is caused by the viscosity of the fluid and the velocity gradient between the fluid and the solid surface. The magnitude of the wall shear stress determines the amount of momentum transfer between the fluid and the solid surface and is an important factor in the analysis of fluid flow in many practical applications, especially on the SC endothelial cells with their basement membrane. The volumetric average shear stress within the intertrabecular spaces and giant vacuoles was found to be approximately 3.2 Pa, whereas in SC, it was observed to be as high as 8.5 Pa. However, caution must be exercised when interpreting the data from regions proximal to the scleral boundary condition due to potential inaccuracies, and thus, these results are not included in our discussion.

## 4. Discussion

The hydrodynamics of the aqueous humor in the JCT and inner wall endothelial cells of SC with their basement membrane is a complex phenomenon that plays a critical role in regulating IOP and maintaining ocular homeostasis [13,25,36,37]. Understanding the flow dynamics in this region is essential for elucidating the underlying mechanisms of glaucoma, a leading cause of irreversible blindness [51,52]. In particular, the JCT and SC regions are known to be the primary sites of resistance to aqueous humor outflow in the trabecular meshwork [9], and abnormalities in the flow dynamics within these regions can lead to elevated IOP and subsequent optic nerve damage. It has been shown that the hydrodynamics of aqueous humor in the JCT and inner wall endothelial cells of SC with their basement membrane is highly complex, with flow patterns and shear stresses that are strongly influenced by the microstructure and geometry of these tissues. It has been hypothesized that changes in the structure and function of these tissues, as well as alterations in the hydrodynamics of aqueous humor, may contribute to the elevated IOP observed in POAG [53]. FSI has emerged as a powerful tool for studying the hydrodynamics of the aqueous humor in these regions, enabling researchers to investigate the effects of various physiological and pathological factors on the flow dynamics and tissue biomechanics. Such studies have the potential to lead to a deeper understanding of the pathophysiology of glaucoma and to guide the development of more effective therapeutic interventions.

The stress and strain exerted on the giant vacuoles with open pores reached levels of 0.8 kPa and 25%, respectively, as illustrated in Figure 4. However, previous studies have established that the plasma membrane can rupture when stretched by 5% or less [54]. The IOP elevation of 22 mmHg resulted in a 50% stretch in the cells of the outflow pathway [55]. Herein, the maximum strain of 25% was observed in the IOP of 7 mmHg, which looks proportional compared to Grierson’s results [55]. Unfortunately, the elastic material model utilized cannot fully account for tissue failure. Consequently, the extensive stresses and strains that the giant vacuoles’ cellular membrane underwent could result in tissue failure or even rupture of the vacuoles themselves. It is possible to speculate that the membrane’s relatively large stretching, induced by such high stresses and strains, may lead to tissue failure or disruption of the giant vacuoles. The ability of giant vacuoles to withstand high aqueous humor pressures may vary depending on their geometry, particularly the thickness of their walls, and their connections with the JCT cells and matrix. If the wall thickness is not adequate, the vacuoles may be more likely to fail or be disrupted biomechanically.

It should be noted that for Model #2 in Figure 3, some of the deepest open spaces were also set to 0 mmHg (the component with green color). This was completed to evaluate the effect of some of the outflow resistance residing beyond the SC inner wall basement membrane 3–5 µm into the JCT. The assignment of 0 mmHg to the inside of giant vacuoles was chosen to simplify the computations and interpretations. The findings from Model #2, as shown in Figure 3, indicate that the regions immediately surrounding the SC inner wall endothelial cells with their basement membrane are more compressed compared to Model #1, which did not show this compression. In Model #1, the open spaces with a pressure of 0 mmHg were situated closer to the SC inner wall endothelial cells with their basement membrane. This observation is consistent with the findings of Hann and Fautsch [56], who demonstrated that an increase in pressure causes the SC inner wall endothelial cells with their basement membrane to be pushed out into SC, leading to a significant enlargement or deformation of the open spaces within the JCT region. In future analyses, the pressure value will be adjusted between 7 and 0 to account for the fluid resistance of the SC cells themselves.

Various studies have reported different numbers of pores in the inner wall endothelial cells of SC as ~33 [57], ~62 [58], and ~292 [30], each with a diameter of about 1.3 µm [28]. Different publications have reported varying numbers of I-pores, as they have used different techniques to count them. Given an aqueous outflow rate of 2.5 µL/min, the resulting velocity in the inner wall endothelial cells of SC with their basement membrane will be 0.95, 0.50, 0.10 m/s (*Q = VA*, *Q* is the rate of the aqueous humor, *A* is the area of all the pores (summation of area in each single pore), and *V* is the aqueous humor velocity). However, in this study, we calculated the aqueous humor velocity based on the active biomechanical interaction between the elastic JCT/SC complex and the incompressible aqueous humor, taking into account the tissues’ mechanical properties. This approach differs from previous calculations based solely on pore area and number [59]. Our models were reconstructed based on the assumption of approximately 292 I-pores [30], which theoretically results in an aqueous humor velocity of 0.1 m/s, which is consistent with the average velocity obtained using FSI (~0.13 m/s) (Figure 5). It should be noted that in Swain’s study [30], the eyes were perfused at 15 mmHg while herein in the experiments and simulations the eye was perfused at 7 mmHg. The velocity of the aqueous humor in the basement membrane of the inner wall of SC was found to be lower when compared to the open spaces of the JCT, resulting in the formation of small eddies near the luminal side of the inner wall of SC (Figure 5). This phenomenon is similar to what was observed in a previous study [13]. The Supplementary Videos display the dynamic velocity vectors of the JCT/SC complex as the aqueous humor pressure increases. The presence of enlarged open spaces with lower aqueous humor velocity upstream of Models #1 and #2 indicates that the resistance is situated within a narrow region of 1–2 μm. This region comprises the SC inner wall endothelial cells with their basement membrane [9,56]. This is a significant finding because it supports the concept that the outflow resistance is not distributed throughout the greater JCT, but rather is confined to the deepest 1–2 μm of this region. This localization of resistance to such a thin area drastically alters our understanding of its nature and the molecular components responsible for it. It implies that the resistance is not a property of the entire ECM of the JCT but instead is exclusively associated with the SC inner wall endothelial cells and their basement membrane.

The average theoretical shear stress of ~0.1–1 Pa through the SC height of ~25 µm was reported using an idealized elliptical cross-section [60,61]. Our FSI results showed the volumetric average shear stress of ~3.2 Pa, through the 20 µm JCT to the SC inner wall (Figure 6). Ethier and Stamer [60,61] used a theoretical approach, so the active biomechanical interaction of the outflow tissues and the aqueous humor has not been taken into account. This difference in approach could explain why our results indicate higher shear stress in the JCT and SC inner wall basement membrane. Herein, the volumetric average shear stress in SC is 8.5 Pa. The high shear stress in this area may be due to the fact that the region that flows leaves the SC basement membrane has an open boundary condition, a non-reflective boundary condition, allowing the flow to exit the SC basement membrane with minimal resistance. Including the collector channels and aqueous veins in future modeling studies could be beneficial, as they would introduce additional resistance along the path of the aqueous humor. However, in most individuals, this would likely be a few mm Hg at most, and from the model standpoint, raising all pressures equally wouldn’t affect our results.

There is extensive evidence showing that the size of true giant vacuoles increases with higher IOP [14,62,63]. In rhesus monkey eyes, measurements have demonstrated a 50% increase in giant vacuole width and a 37% increase in length between IOP levels of 8 and 15 mmHg [55]. Increasing pressure from 2 to 6 mmHg increased giant vacuole height by 7 ± 3 µm [64]. Our findings suggest that the cellular membrane of the giant vacuoles experiences significant stresses and strains due to the pressure gradient across the inner wall of SC. This effect is particularly pronounced in giant vacuoles with open pores as illustrated in Figure 4 (right side of the models). However, the basement membrane appears to serve as a control mechanism, helping to mitigate these stresses and strains in the thinner top region of the giant vacuoles, where the cellular membrane is closer to SC (Figure 4). In a study involving rhesus monkeys, Grierson reported an increase of 2.4 µm and 5.2 µm in the width and length of giant vacuoles at an IOP of 15 mmHg [55]. Our results showed a 45% increase in width and 34% increase in length of the giant vacuoles, which translated to an increase of approximately 4.2 µm and 3.0 µm, respectively. In this study, the width and length of giant vacuoles were measured at two distinct pressure levels to assess their physical properties and response to changes in pressure. The giant vacuoles’ size, including the length and width, herein related to the deformation of these vacuoles due to the aqueous humor pressure elevation. The comparative analysis of vacuolar dimensions provides valuable insights into the underlying biological mechanisms involved in cell expansion and contraction, osmoregulation, and water balance. The large stresses and strains in the cellular lining of a giant vacuole in our models may have caused them to collapse, while in the aforementioned study, the lack of tensile strength in the material model prevented the modeling of this phenomenon.

Lai et al. [29] investigated whether cellular connectivity between SC inner wall (IW) endothelium, and JCT, and between inner wall endothelial cells, plays a role in giant vacuole and pore formation by comparing perfusion-fixed (15 mm Hg) and immersion-fixed (0 mm Hg) eyes. They found that the mean cell width in non-nuclear areas is significantly narrower in perfusion-fixed eyes (3.90 ± 0.41 μm) than in immersion-fixed eyes (8.01 ± 0.63 μm). Herein, in our simulations, the geometry of the JCT/SC complex was reconstructed based on the perfusion-fixed eyes at the pressure of 7 mmHg. Thereafter, we applied an additional 7 mmHg to the geometry through the FSI simulation resulting in having our final models at the “nominal pressure” of ~14 mmHg. Thus, we can fairly assume that the resultant geometry through FSI simulations (~14 mmHg) should be relatively close to what was reported by Lai et al. [29] at a pressure of 15 mmHg. FSI results in nucleated cells on average showed the width decreasing by ~1.8 µm in both models #1 and #2 which is within the range of reported values in Lai et al. [29] considering the comparison between 14 mmHg and 7 mmHg in FSI simulations (7 mmHg elevation in pressure). When it comes to the mean volume of giant vacuoles, Lai et al. [29] showed a significantly larger volume in perfusion-fixed eyes (144.98 ± 47.65 μm^3^), compared to the immersion-fixed eyes (27.18 ± 7.09 μm^3^) considering the pressure difference of 15 mmHg between two geometries [29]. While our FSI simulation did not include the entire volume of the giant vacuoles in the model, we fit a circle to each giant vacuole and we used the average resultant diameter to reconstruct a sphere to calculate the volume based on the MATLAB code described in our prior publication [65], and compared it to Lai et al. [29]. The FSI simulations on average showed ~81 μm^3^ change in the giant vacuole volume for 7 mmHg pressure elevation due to an active biomechanical interaction between the JCT/SC complex and aqueous humor, which is in good agreement with Lai et al., [29].

Through the use of computational analysis, this study has identified localized high-stress and strain regions that may cause variations in the levels of strain and stretching experienced by cells in different areas. Specifically, mechanical stretch and distortion can trigger a response in outflow pathway cells that leads to the adjustment of ECM turnover and outflow resistance. This response helps to maintain the homeostasis of IOP [9,15,19]. The identification of these high-stress and strain regions and their effect on cellular behavior provides important insights into the regulation of IOP. The distribution of stresses and strains in the inner wall endothelial cells of SC with their basement membranes can indicate which regions are more resistant to outflow and therefore contribute more significantly to overall resistance compared to other regions. In other words, areas of higher stress and strain may be indicative of regions that are more critical to the overall stability and function of the inner wall endothelial cells of SC with their basement membranes. This information may prove useful in the development of new treatments for ocular diseases, such as glaucoma, which are characterized by IOP dysregulation. Overall, this study underscores the significance of mechanical forces in influencing cellular behavior and physiological processes.

The results of this study have shown that, in addition to segmental outflow (the tissue in this study is from a high-flow region), there is also a driving potential for significant micro-scale outflow segmentation. This segmentation occurs at irregular intervals of 50–150 µm and results in alternating high and low fluid drainage rates in different areas of the outflow pathway [4,5]. The discovery of this micro-scale outflow segmentation highlights the complex nature of the outflow pathway and suggests that a more nuanced understanding of its structure and function is required to develop effective treatments for glaucoma. This information may be particularly useful for the development of targeted therapies that can address specific regions of the outflow pathway, leading to improved regulation of intraocular pressure. The current understanding is that segmental outflow regulation in the outflow pathway is complex and relies on unknown regional mechanisms, assuming uniform pressure and stresses around the circumference of the JCT. However, our study has identified micro-scale regions of varying stress and strain within the outflow pathway. This suggests that the regulation of IOP may be triggered locally and as needed, rather than relying on a separate complex segmental mechanism. These findings introduce an exciting new regulatory concept that warrants further investigation. By understanding the specific micro-scale regions of high and low stress and strain within the outflow pathway, we may be able to develop targeted therapies to address specific areas of the pathway and improve IOP regulation. Overall, this study highlights the importance of considering the micro-scale variations in stress and strain within the outflow pathway, rather than assuming uniform pressure and stresses. Developing a better understanding of the specific regions in the eye that play a major role in outflow resistance can have significant implications for the treatment of ocular diseases that are associated with IOP dysregulation. Targeted drug delivery via nanodrug carriers could be utilized to selectively deliver drugs to these regions and enhance their effectiveness in reducing IOP.

### Limitations

First, the current study involved only one healthy donor eye for both the experimental and the computational analyses; thus, more sections and more samples are necessary to represent the complete spectrum of geometries in individuals’ outflow tissues. The main aim of this research was to establish a workflow that utilizes precise, eye-specific SBF-SEM images to simulate the biomechanics of the inner wall endothelial cells of SC with their basement membrane and JCT. In subsequent studies, a greater number of human eyes, including both healthy and glaucoma eyes, with diverse outflow tissue geometries, will be included.

Second, the current study specifically concentrated on modeling the high-flow region of the flow within the TM structure, while the low-flow region was not considered [3,66]. In addition, we selected regions that did not exhibit clear SC basement membrane discontinuities, which are thought to be the point of aqueous humor movement through the outflow resistance. However, in future research, both low- and high-flow regions will be simulated to compare the hydrodynamics of the aqueous humor in the JCT and inner wall endothelial cells of SC with their basement membranes and investigate the biomechanical response of these tissues.

Third, the final FE model of the JCT/SC complex in this study was reconstructed with an element edge length of approximately 0.25 µm, despite the SBF-SEM images having a pixel size of 0.0101 µm. While the image-to-mesh algorithm generated a 3D FE mesh with an element edge length of 0.0101 µm, the sheer number of elements for each slice of the SBF-SEM image, which was approximately 20 million, made it impractical to work on the model with our 112-core workstation, even though it had substantial computational power. Consequently, we only used two sets of images for each model, whereas it would have been preferable to have more sets of images to develop an interconnected network of structures within the JCT and SC inner wall basement membrane. In future studies, we will seek a computational solution that enables us to add a higher number of slices to the model, thereby allowing for interconnectivity between intertrabecular spaces across the thickness of the JCT/SC complex.

Fourth, in this study, only the JCT and inner wall endothelial cells of SC with their basement membrane were modeled, even though the conventional aqueous outflow pathway includes the TM, JCT, and inner wall endothelial cells of SC with their basement membrane. However, in normal human eyes, the JCT and the immediate vicinity of the SC inner wall basement membrane are the primary sites of aqueous humor outflow resistance, which is why modeling only these two regions is sufficient for the study’s purposes. Additionally, incorporating the TM into the model would drastically increase the number of elements [67,68], thereby creating computational difficulties due to our limitations in computational power.

Fifth, in this study, the JCT and SC inner wall were modeled as linear elastic, despite previous evidence indicating their hyperviscoelastic behavior due to the directional distribution of collagen fibrils in the ECM of the JCT [12,25,35,36,37]. Nonetheless, the study’s primary goal was to investigate the biomechanics of the JCT and inner wall endothelial cells of SC with their basement membrane using high-fidelity, high-resolution, eye-specific SBF-SEM images. To further explore this aspect, future studies will use a larger cohort of healthy and glaucoma eyes and incorporate complex hyperviscoelastic material models.

Sixth, this study did not consider the formation of pores in the giant vacuoles that result from the tissue’s mechanical failure when the applied tensile and compressive load due to an IOP elevation exceeds the tissue’s yield stress. This is because the study used a simple linear elastic material model that does not account for the material’s tensile or compressive strength. In future studies, if information about the tensile and compressive yield stress of the inner wall basement membrane of the Schlemm’s canal becomes available, it will be included in the simulations.

Seventh, similar to vascular endothelial cells, an ECM layer of proteoglycans and glycoproteins known as glycocalyx with an average thickness of 52–166 nm [69] is present in the human conventional aqueous outflow pathway covering the TM, JCT, and SC cells, some of the I-pores in the inner wall endothelial cells of SC as well as the inner membrane of giant vacuoles [60,61,69,70,71,72]. The glycocalyx layers functioning as sensors of fluid shear stress [73,74,75] are responsible for aligning endothelial cells in the direction of flow [76,77,78]. Additionally, versican plays a critical role in outflow resistance by organizing other ECM components to facilitate and regulate open flow channels in the JCT [9,79]. Versican is a large, chondroitin sulfate-substituted proteoglycan that binds hyaluronan through its G1 domain [80]. It is worth noting that in the concurrent study, neither the glycocalyx layer nor versican was included in the model and the tissue preparation is known to collapse the glycosaminoglycan sidechains. This omission could have affected the calculated outflow resistance in the JCT and inner wall endothelial cells of SC with their basement membranes. While our previous numerical study has indicated that the glycocalyx in the outer trabecular beams of the aqueous outflow pathway does not significantly impact the wall shear stress and velocity of the aqueous humor [40], the role of versican in outflow resistance within the SC basement membrane requires further investigation.

Eighth, during the experiment, the JCT/SC complex was perfusion-fixed and imaged at a pressure of 7 mmHg. However, during the modeling phase, we pressurized the tissues to 7 mmHg using FSI simulation, which may cause an overestimation of the deformations and strains in the tissues. That means we applied an additional 7 mmHg to the model that may give us the geometry at the aqueous humor pressure of 14 mmHg. Although it would have been preferable to have the tissues at 0 mmHg pressure, doing so could lead to the collapse of giant vacuoles and smaller intertrabecular spaces, which may affect the 3D reconstruction of the JCT/SC complex. In future studies, we plan to use numerical techniques to calculate the geometry of the JCT/SC complex at a pressure of 0 mmHg.

Ninth, the uveal outflow tract, specifically the uveoscleral pathway, holds a distinct role in the dynamics of intraocular pressure regulation. While our focus primarily revolves around the conventional outflow pathway, it is important to acknowledge the uveoscleral route’s pressure-insensitive nature and its limited contribution of less than 30% to the overall outflow facility in humans. By design, our model excludes this pathway and any potential downstream resistance stemming from Schlemm’s canal. Consequently, any effects or contributions originating from these neglected aspects would indeed remain unapparent in our findings. While our study concentrates on the dominant mechanisms governing normal outflow, the uveal outflow tract undoubtedly plays a nuanced role that could influence results in a comprehensive analysis. Future investigations might delve deeper into the interplay between the conventional and uveal pathways, potentially enriching our understanding of intraocular pressure regulation.

Finally, this study did not model the hydraulic conductivity, despite evidence showing that it contributes to approximately 10% of the total outflow resistance [22,81]. However, the primary objective of this study was to model the interaction of the aqueous humor with the JCT and inner wall endothelial cells of SC with their basement membranes, utilizing high-fidelity, high-resolution, eye-specific SBF-SEM images. Thus, the geometry of the JCT and inner wall endothelial cells of SC with their basement membranes was the focus of this study, with future studies intending to incorporate the hydraulic conductivity of the tissues into the model.

## 5. Conclusions

The fluid–structure interaction has emerged as a powerful tool for studying the hydrodynamics and biomechanics of the inner wall endothelial cells of SC with their basement membrane and JCT. FSI simulations enable researchers to model the complex interaction between fluid flow and the deformable basement membrane structure. By incorporating the mechanical properties of the cellular membrane into the simulation, FSI can reveal the stresses and strains that the JCT and SC inner walls experience under different physiological conditions. In this study, a high-fidelity 3D finite element model of the JCT and inner wall endothelial cells of SC with their basement membrane was reconstructed using serial block-face scanning electron microscopy images. The interaction of the SC/JCT complex with the aqueous humor was modeled using the FSI MM-ALE method, and the resultant hydrodynamics of the aqueous humor as well as the biomechanics of the JCT/SC complex were investigated. Notably, larger stresses and strains were observed in the cellular membrane of giant vacuoles, particularly those with open pores. Moreover, funneling was detected through the pores into SC. The average resultant velocity and wall shear stress in the SC inner wall basement membrane were found to be well within the range of reported values in the literature. FSI helped us to map the velocity vectors across the SC inner wall and the open spaces in the JCT as a function of aqueous humor pressure elevation and time. These findings may have significant implications for our understanding of aqueous humor hydrodynamics in healthy and glaucoma eyes in both low- and high-flow regions. From the identification of these regions, we can postulate a built-in mechanism of regional triggering of the IOP homeostatic response where it is most needed. Indeed, FSI can illuminate the pathophysiology of ocular diseases, such as glaucoma, which are characterized by elevated IOP due to impaired aqueous humor outflow through the TM.

## Data Availability

The raw/processed data required to reproduce these findings cannot be shared at this time as the data are part of an ongoing study.

## Supplementary Materials

The following supporting information can be downloaded at: https://www.mdpi.com/article/10.3390/bioengineering10091038/s1, Video S1: Velocity map of the flow across model #1; Video S2: Velocity map of the flow across model #2.
